# The Influence of Entrepreneurship on the Innovation Path of Cultural Enterprises Under the Background of Digital Transformation

**DOI:** 10.3389/fpsyg.2022.892348

**Published:** 2022-06-09

**Authors:** Siting Liu, Yong Zhou, Changlin Wang, Yufan Yu

**Affiliations:** ^1^School of Marxism, Weifang University, Weifang, China; ^2^School of Political Science and Law, Weifang University, Weifang, China; ^3^School of Economics and Management, Binzhou University, Binzhou, China

**Keywords:** entrepreneurship, corporate innovation performance, questionnaire, correlation analysis, regression analysis

## Abstract

This study aims to explore the impact mechanism of the new dimension of entrepreneurship on the innovation performance of enterprises in the context of digital transformation. The relationship between entrepreneurship and enterprise innovation performance is studied in Beijing, Shanghai, Guangzhou, Shenzhen, and other high-tech enterprises represented by innovative companies. Firstly, the correlation hypothesis between variables is proposed through the induction and summary of relevant data and academic achievements. Statistical data is collected by means of a questionnaire survey. The content of the questionnaire includes three dimensions: 1. collective innovation, risk-taking, and integrity; 2. the survival performance and growth performance of new ventures; 3. basic information about the enterprise, such as the size, age, type, and classification of different Internet and the location. Statistical Product and Service Solutions (SPSS) and Advanced Mortar System (AMOS) statistical software are used to carry out statistical and correlation analyses of the valid questionnaires. Finally, the proposed hypothesis is verified through regression analysis. To sum up, the main conclusions are: the correlation coefficients between innovative spirit, adventurous spirit, integrity, and enterprise survival performance are 0.401, 0.426, and 0.393, respectively, which are positive correlations. The correlation coefficients between innovative spirit, adventurous spirit, integrity, and enterprise survival performance are 0.434, 0.367, and 0.536, respectively, and there is a significant and positive correlation. It shows that entrepreneurship and its three dimensions have a significant positive impact on enterprise entrepreneurial performance. The research examines the logical relationship and influence mechanism between the entrepreneurial spirit of entrepreneurs and the innovation performance of enterprises, which has a certain guiding role in the management practice of innovative enterprises in China.

## Introduction

The most important thing in strategic management research is how to make the enterprise have a higher level of performance. However, at present, the changing trend of the global economy is more complicated. In today’s unpredictable global market economy, if companies want to gain a larger market share, they can only continue to innovate. Innovation is the core factor for companies to survive in the market for a long time, and it is also a key tool for companies to improve their own performance. Enterprises must clarify how to innovate and the meaning of enterprise innovation. Only by fully understanding the meaning and significance of innovation performance and exploring ways to improve enterprise innovation performance can enterprises fundamentally grow. With the rapid development of the digital economy in recent years, and the impact of corona virus disease 2019 (COVID-19), digital economy technology has become an important driving force for economic development, and more and more enterprises are inseparable from innovation, transformation, and development digital support. Entrepreneurship plays a certain role in the improvement of corporate innovation performance (Mikhaylova et al., 2021). However, in the new economic era, entrepreneurship has new connotations and new forms of expression, and it is necessary further to explore the relationship between entrepreneurship and corporate innovation performance.

At present, scholars have put forward different views on the relationship between entrepreneurship and corporate innovation performance. Entrepreneurship can promote enterprise innovation performance. To interpret the relationship between the two from the perspective of entrepreneurship characteristics, that is, entrepreneurship has a positive impact on corporate financial performance, and the positive impact is very significant in the fierce environmental competition (Jeong, 2021). Additionally, some scholars have found that entrepreneurship can promote technological innovation, product innovation, and market innovation of enterprises and make the relationship between the three closely linked to promoting product research and development of enterprises. Interpret the relationship between the two from the perspective of organizational learning. That is, corporate entrepreneurship has a positive impact on new product innovation performance, and organizational learning has a mediating effect on the relationship. In addition, the moderating effect of knowledge acquisition on the relationship between entrepreneurship and corporate innovation performance is negative, and the amount of knowledge acquisition depends on the level of corporate knowledge resources (Kusa et al., 2021). Interpret the relationship between the two from the perspective of organizational structure. That is, entrepreneurship can promote the financial performance of enterprises. Especially in organic-style organizational structures, entrepreneurship, as corporate behavior, is intertwined with the corporate vision, strategic goals, structure, and operations and has a direct and positive impact on performance (Wu et al., 2022).

At present, the research on the impact of entrepreneurship on enterprise innovation performance has been relatively in-depth and extensive. However, the influence of each dimension of entrepreneurship on the innovation performance of enterprises is unknown. Based on sorting out the existing research results, this study innovatively explores the influence of various dimensions of entrepreneurship on the innovation performance of enterprises. Firstly, correlation hypotheses between variables are proposed. Statistical data are collected through the questionnaire survey method. Statistical software is used to carry out statistical and correlation analysis on valid questionnaires, including verifying the reliability and validity of the questionnaire and the analysis of the correlation between variables. Finally, the proposed hypothesis is verified by regression analysis. The conclusion provides a reference for revealing the relationship between entrepreneurship and innovative enterprise performance and further enriching the theory of entrepreneurship in the local context.

## Research Theoretical Basis and Questionnaire Design

### Entrepreneurship Connotation and Measurement

**Figure 1:**
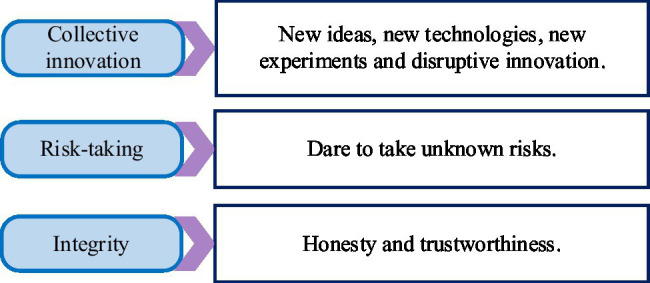
Dimensions and connotations of entrepreneurship.

For a long time, the role of entrepreneurship in innovative enterprises has been the focus of extensive discussion in academia. Many kinds of literature and research conclusions show that entrepreneurship is one of the important factors for enterprises to gain a competitive advantage and generate performance. Entrepreneurship has a positive impact on the organization and development of startup companies or mature enterprises. The dimension of entrepreneurship and its connotation is shown in Figure 1.

One of the elements that form the core of entrepreneurship is collective innovation. Whether the entrepreneurial team is innovative or creative is the key factor to measure the stable and sustainable development of entrepreneurship. As the American management, Peter F. Drucker proposed, in entrepreneurship, the most important thing is team innovation. A critical step in managing a business is the entrepreneur’s economic and behavioral risk-taking initiative. The focus of entrepreneurship is to identify and explore market opportunities that have never been discovered before. Through creative arrangement and combination of original factors of production and resource allocation, continuous organizational performance can be achieved through launching new services or products, developing new markets, and exploring and expanding new users (Wu and Wu, 2017; Zhang and Cao, 2020; Khazaei, 2021).

Entrepreneurship is essentially the collective innovation and continuous pioneering spirit of an organization at the team level, which is also an important force in promoting economic development. The characteristics of collective innovation, risk-taking, and integrity are the main manifestations of entrepreneurship. Entrepreneurship is a dynamic resource transformation and allocation mechanism. Its collective innovation, adventure, and integrity will be constantly adjusted to the change in the external environment. The integration of internal and external resources of the enterprise has become its own unique place.

As the world pays more and more attention to the entrepreneurial economy, so does the academic literature on entrepreneurship. There are also many theoretical studies in this field, but the understanding and measurement of entrepreneurship have not yet been unified (Prasetyo and Kistanti, 2020). The methods that are often mentioned are as follows: entrepreneurship needs to focus on the identification, evaluation, and development of the feasibility of entrepreneurial opportunities to distinguish entrepreneurship from other research in the field of entrepreneurship. In addition, some scholars believe that the measurement dimensions of entrepreneurship are diversified, but the main indicators for measurement are innovation and competitiveness. Entrepreneurship can also be assessed in innovation, pioneering, risk-taking, opportunity acumen, and tolerance (Schalkwyk, 2020).

Entrepreneurship is an important factor in promoting the development and growth of new ventures. All entrepreneur groups have essentially the same entrepreneurial spirit and entrepreneurial values. These spirits and values are expressed through the entrepreneurial activities and behavioral attitudes of entrepreneurs or enterprises. Entrepreneurship is mainly characterized by three dimensions: collective innovation, risk-taking, and integrity.

### Overview of Enterprise Innovation Performance

#### Concept and Connotation of Innovation Performance

Performance is the operating results shown by an enterprise during its operation, and it is the only standard used by an enterprise to measure its operational level and determine the core issues of strategic management. It is a relatively broad and complex concept, it is the ultimate starting point of all strategic management theories, and it is also the most important indicator for enterprises to express their management and development capabilities in the process of market development. In addition, performance can be analyzed from both the results perspective and the process perspective. From the perspective of results, performance is an indicator of quantitative work. From the perspective of process analysis, the performance demonstrates enterprise capability. So far, since there is no unified standard to determine the connotation of enterprise performance, the measurement and measurement of performance still needs many models as support.

Innovation performance simply refers to the behavioral capabilities and characteristics of an enterprise in the process of product innovation. Innovation is not only the most important symbol for an organization to distinguish itself from other organizations in the process of market development but also an important symbol for an enterprise to highlight its economic strength in the process of market development (Qian et al., 2018; Wu and Song, 2019; Usai et al., 2021).

**Figure 2:**
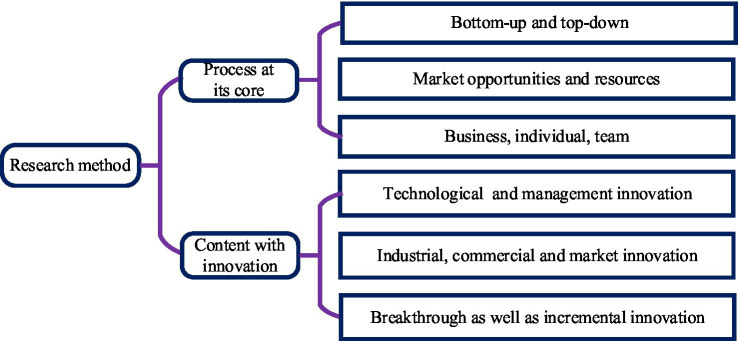
The specific connotation of innovative research methods.

The concept of innovation is highly comprehensive, and many scholars have adopted the extension and connotation dimension division methods to study the concept of innovation. In general, innovative research methods can be divided into two broad categories, as shown in Figure 2.

In Figure 2, the content of innovation performance is also very rich due to the inconsistency of the perspective of performance definition. So far, there is still no unified definition of innovation performance. Combined with the innovation performance theories of most scholars, the definition of innovation performance can be divided into two broad senses and narrow senses. Innovation performance in a narrow sense is the process of introducing a market system by an enterprise. It is also the process of introducing a higher level of technology to promote the development of its own products. The innovation performance in a broad sense is that the process of implementing the ideas and concepts of the enterprise includes the market input link of the product and the technological innovation link of the product.

The general meaning of innovation performance is the efforts made by the enterprise to obtain longer-term development and the results obtained in the process of product production. In the fierce market competition environment, more and more enterprises have begun to pay attention to the issue of product innovation. Product innovation has also grown rapidly in recent years as an important part of the core development of enterprises. Regardless of the perspective, the result of formal or process or technological innovation is reflected in the development of products.

#### Measurement of Innovation Performance

**Figure 3:**
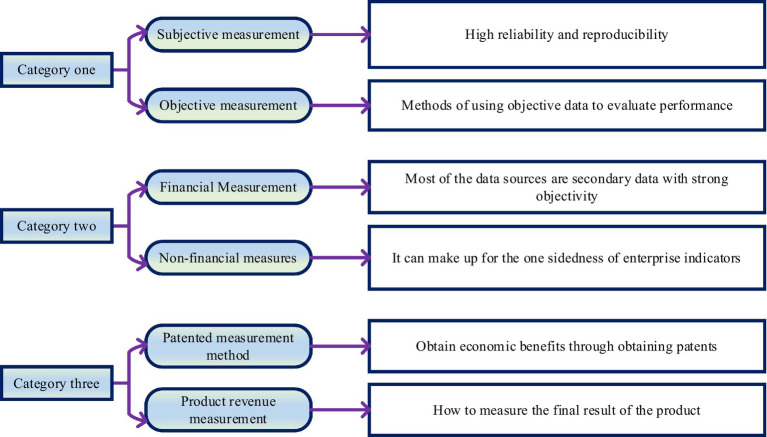
The specific meaning of each measurement method.

When researchers study how innovation performance is measured, they will incorporate innovation into the field of management. They always explore innovation performance as a result of product production. From another perspective, enterprise innovation performance is the internal management of enterprises. In the preliminary research stage of enterprise innovation performance, most scholars pay special attention to the whole process and think that the innovation performance of enterprises is the change in quantity. However, in the subsequent development process, with the continuous enrichment of the concept of innovation performance, people realized that it is impossible to measure innovation performance simply by quantity, so they began to explore a more rigorous way to create a comprehensive and systematic structure. People constrain performance in broader concepts to diversify tools for measuring innovation performance (David et al., 2021). In the process of measuring enterprise innovation performance from different perspectives, researchers have selected many methods and methods. After these methods and methods are summarized, they can be divided into three measurement methods. The specific meaning of each measurement method is shown in Figure 3.

In Figure 3, the methods of measurement are varied. Different measurement indicators correspond to different measurement methods. Even if the same category is measured, different measurement methods will be selected due to different calculation methods and measurement indicators. In the process of innovative performance measurement, it is necessary to determine a reasonable measurement method according to the actual measurement needs and then create a comprehensive theoretical framework for education based on the measurement method.

### The Impact of Entrepreneurship on Corporate Innovation Performance

Entrepreneurship is an important factor for the survival of entrepreneurial enterprises and the core performance of whether they are competitive in the market. The startup is divided into two categories: a positive and innovative spirit and a lack of innovative spirit. Innovative companies have a clear industry reputation in the market. This reputation leads to positive feedback, such as high user loyalty and satisfaction. If a startup has a strong entrepreneurial spirit, then the company has a keen market sense and can respond quickly and efficiently to fast-moving changes. This capability strongly safeguards the company’s competitive advantage and enables the startup company to have excellent growth and financial performance in the future.

The logic of the positive correlation between entrepreneurship and innovative enterprise performance can be summarized in the following two aspects. Firstly, innovative, pioneering, and adventurous entrepreneurship can make startup companies have obvious competitiveness and bring competitive advantages at the operational level. Secondly, innovative, pioneering, and risk-taking entrepreneurship can make startups more resilient to market changes. This spirit makes it easier to make quick and efficient decisions in the face of changing consumer and market landscapes. Therefore, entrepreneurship has a very positive effect on startup companies to increase market share, increase sales profits, guide market prices, expand market distribution channels, and other indicators (Kim, 2017; Umair et al., 2018; Young and Tae, 2019).

### Research Route

**Figure 4:**
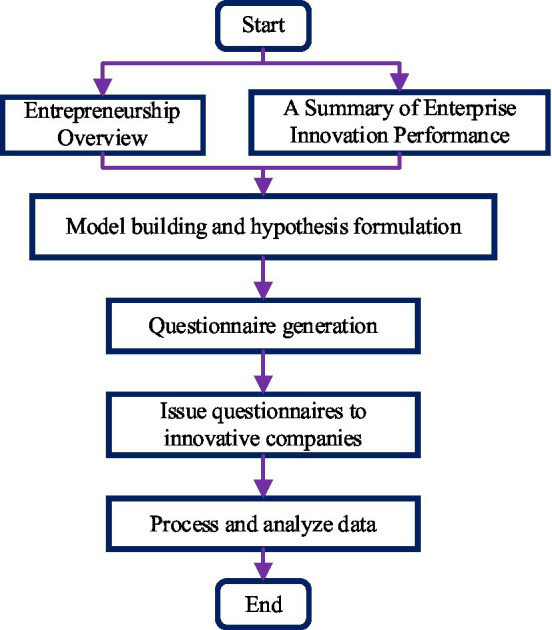
Research framework.

Firstly, based on the existing academic literature, questionnaires measuring entrepreneurship and startup performance are completed. Then, according to the small-scale pre-survey results, some items are modified and adjusted to obtain a survey questionnaire that is more suitable for the specific research background in China. The research idea is shown in Figure 4.

#### Questionnaire Design

The design of the survey questionnaire is mainly divided into two steps: firstly, the content of the survey questionnaire is determined. The research content includes variables designed, such as entrepreneurship is divided into three dimensions: collective innovation, risk-taking, and integrity. The performance of new ventures includes survival performance, growth performance, and so on. Additionally, the survey content also includes basic information about the company, such as company size, company age, different types of Internet classifications, and the region where they are located. Secondly, the format of the survey questionnaire is determined. Several indicators describe each variable in the survey questionnaire. The subjects are required to answer these questions one by one when filling out the questionnaire. One stands for “strongly disagree,” five stands for “strongly agree,” and the others are in the middle (Edem et al., 2020; Mehboob and Kauermann, 2021).

#### Samples and Data

Beijing, Shanghai, Guangzhou, and Shenzhen are the main areas for research, where technological innovation and entrepreneurship activities are more active. The innovative companies selected for the questionnaire survey have three conditions: 1. the company has been established for at least 6 months and new ventures within 8 years. 2. The enterprise must be an independent company, not a subsidiary or branch of the group headquarters or the parent company. 3. Exclude companies that only have distribution and do not have their own production or technology research and development departments. In order to ensure the authenticity and validity of the data, the interviewees are mainly the founders of the enterprise or the core management members of the entrepreneurial team. They are required to be at least in the middle management positions of the enterprise and above, such as CEO, vice president, marketing, or technical director (Alfredo et al., 2021).

Questionnaire questions are mainly in the form of multiple-choice questions. Questionnaires are distributed and collected for a duration of 3 months. Field research, online filling, email, and other forms are used for distribution. A total of 320 questionnaires are distributed, and 311 are recovered, with a recovery rate of 97.19%. Invalid samples are excluded. There are 300 valid questionnaires, and the effective recovery rate of the questionnaire is 93.75%. Questions with missing content in the questionnaire and with obvious regularity in the scoring of the questions (for example, most of the items are almost completely consistent, etc.) will be regarded as invalid samples and will be eliminated.

#### Selection of Research Variables

**Figure 5:**
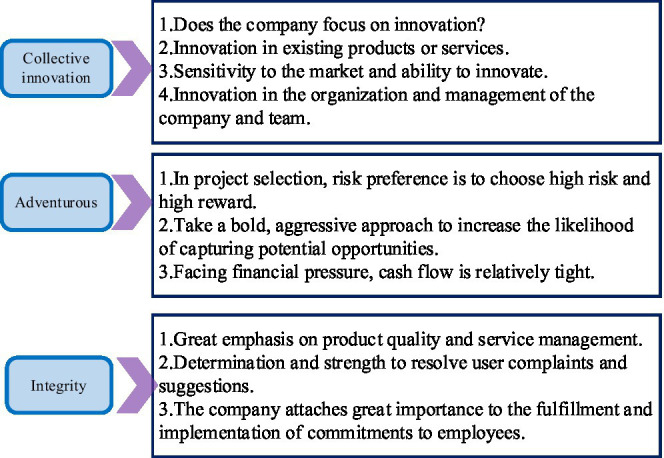
The three-dimensional scale of entrepreneurship.

Entrepreneurship is used as an independent variable. Based on referring to existing scales, entrepreneurship is measured from three dimensions of collective innovation, risk-taking, and integrity by summarizing and arranging relevant mainstream literature. The three-dimensional scale is shown in Figure 5.

**Figure 6:**
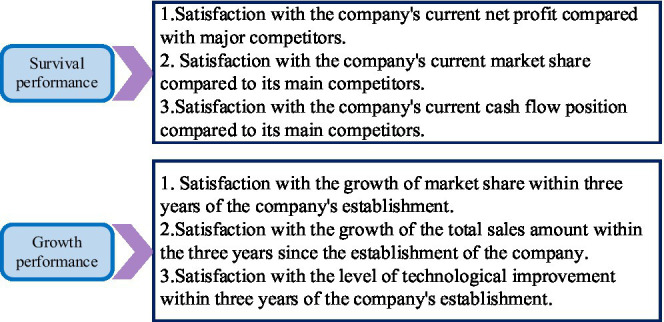
Enterprise innovation performance scale.

In Figure 5, corporate innovation performance is used as a dependent variable. The measurement of enterprise innovation performance can be recognized and measured from different perspectives. This perception and measurement can be influenced by the level of analysis and the differences in strategy. Usually, two methods of subjective evaluation and objective statistics are used to evaluate the performance of new ventures. Both methods have their pros and cons. The two dimensions of survival performance and growth performance are used to evaluate and measure enterprise innovation performance. The enterprise innovation performance scale is shown in Figure 6.

#### Reliability and Validity Analysis and Correlation Analysis of Variables

Statistical Product and Service Solutions (SPSS) and Advanced Mortar System (AMOS) statistical software are used to carry out statistical and correlation analysis on valid questionnaires, including verifying the reliability and validity of the questionnaires and the analysis of the correlation between variables.

##### Scale Reliability and Validity Analysis

*Reliability Test.* Reliability refers to the analysis of the same event under the condition that the research method does not change, and the result does not change, indicating that the survey results have high reliability, so it can also be called reliability analysis (Emerson, 2021). At present, the commonly used reliability measurement index is Cronbach’s alpha, as shown in Eq. (1):

(1) $$\alpha =\left(K/\left(K-1\right)\right)*\left(1-\overset{K}{\underset{i=1}{\sum }}\sigma_{Y^{i}}^{2}/\sigma_{X}^{2}\right)$$

K represents the total number of questions in the questionnaire; $\sigma_{X}^{2}$ represents the variance of the total sample; $\sigma_{Y^{2}}^{2}$ represents the variance of the measurement sample. SPSS 25.0 is used to analyze the data results obtained by the questionnaire survey, and the value of *α* is between 0–1. If 0.9 < *α* < 1, it indicates that the survey results have high reliability; if 0.8 < *α* < 0.9, it indicates that the survey results can be used for research analysis; if 0.7 < *α* < 0.8, it indicates that the survey results are reliable lower, and needs to be modified accordingly.

*Content Validity Analysis.* The content validity of the measurement questionnaire mainly depends on whether the actual situation of question selection is typical and comprehensive. If the questionnaire items are based on theory and adjusted and improved concerning the content of previous similar studies, they can be considered to have good content validity (Molgaard et al., 2021). Content validity is a subjective measurement element. Firstly, the measurement of all variables is formed based on existing academic research and literature. These scales have been used by many researchers and scholars and have been extensively tested. Advanced Mortar System (AMOS) software is used for analysis.

##### Correlation Analysis

*Moderating Effect Test.* The moderating variable means that if there is a relationship between the variables *X* and *Y*, but the relationship between *X* and *Y* is affected by the third variable *Z*, so *Z* is the moderating variable. The role of *Z* between *X* and *Y* is called regulation (Anna et al., 2020). Statistically, the effect of the moderating variable is represented by the product of the two variables, *X* and *Z*, as shown in Eq. (2):


(2) $$Y=\beta_{0}+\beta_{1}X+\beta_{2}Z+\beta_{3}X*Z$$

*β*_0_ represents the adjustment variable; *β*_1_ represents the influence coefficient of X on Y. *β*_2_ Table Z’s influence coefficient on *Y*. *β*_1_and *β*_2_ reflect the size of the main effect. *β*_3_ reflects the magnitude of the regulatory effect. If the coefficient *β*_3_ of the product term is significant, it means that the moderating effect exists. That is, it proves that *Z* is the moderator variable of the main effect.

SPSS software is used to test the moderating effect in the following way. Firstly, the observed values of the independent and moderator variables are normalized (using Z-scores), and a product term is constructed. This is to reduce the problem of multicollinearity among the variables in the regression equation. Then, the control variable, independent variable, moderating variable, and product term are put into the multivariate hierarchical regression model and the dependent variable, in turn, to test the moderating effect, as shown in Eq. (3):


(3) $$Y=\beta_{0}+\beta_{1}X+\beta_{2}Z+\beta_{3}\bar{X}*\bar{Z}$$

*X*, *Z*, *Y* are unnormalized values, $\bar{X}$ and $\bar{Z}$ are normalized values.

## Experimental Results

### Basic Characteristics of the Sample

**Figure 7:**
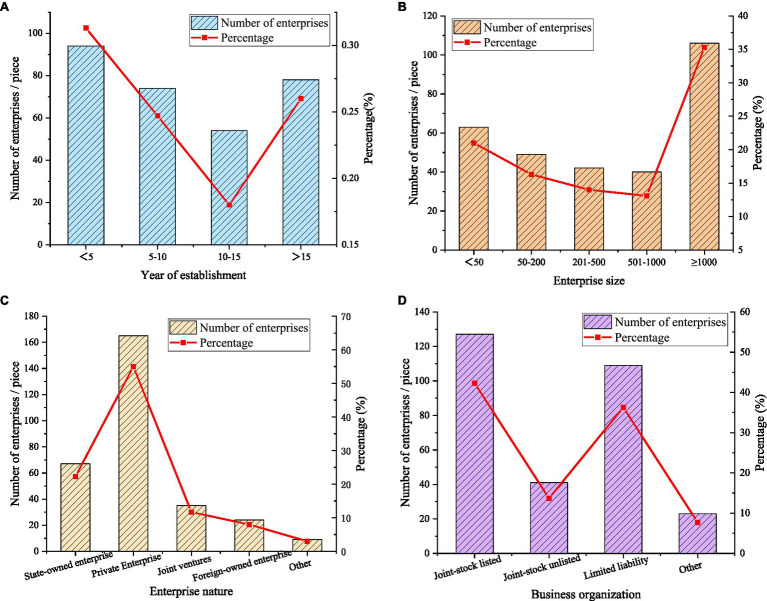
Basic characteristics of the sample. **(A)** Years of the establishment of the enterprise; **(B)** Enterprise scale; **(C)** Enterprise nature; and **(D)** Enterprise organizational form.

The basic characteristics of the sample include the years of establishment of the enterprise, the scale of the enterprise, the nature of the enterprise, and the organizational form of the enterprise, as shown in Figure 7.

In Figure 7A, 31.3% of the companies in this survey have been established for less than 5 years. 24.7% of enterprises are established between 5 and 10 years. 18% of the companies are established between 10 and 15 years. 26% of enterprises are established for 15 years or more. The sample distribution is relatively uniform. In Figure 7B, small and medium-sized enterprises accounted for 64.7%, and large enterprises (1,001 employees and above) accounted for 35.3%. In Figure 7C, the surveyed companies are mainly private companies, accounting for 55%. In Figure 7D, joint-stock listed companies account for 42.3%, and limited liability companies account for 36.3%.

### Questionnaire Reliability and Validity Analysis

**Figure 8:**
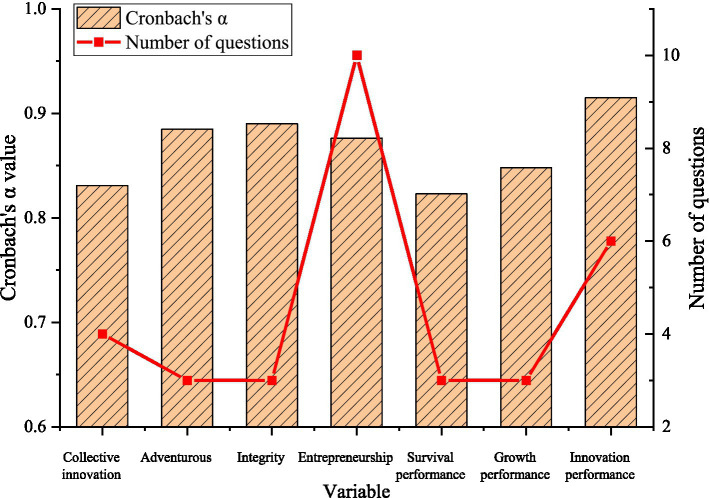
Test results of reliability of variables and dimensions.

Cronbach’s alpha is used as the evaluation index of reliability, and the results obtained by SPSS 25.0 analysis are shown in Figure 8.

In Figure 8, the measurement reliability values of the seven dimensions of the two variables are all greater than 0.8. The comprehensive reliability of entrepreneurship and corporate innovation performance are 0.876 and 0.915, respectively. Therefore, the dimensions of all variables in the measurement scale meet the requirements, and the comprehensive reliability of the two variables has very good internal consistency.

**Figure 9:**
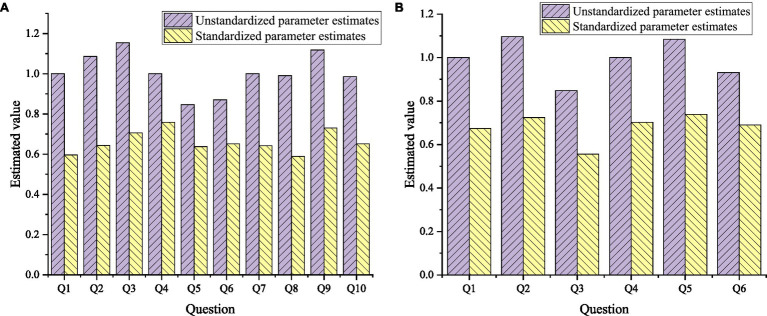
The results of the confirmatory analysis of the scale. **(A)** Entrepreneurship Scale and **(B)** Enterprise Innovation Performance Scale.

AMOS24.0 is used to perform confirmatory factor analysis on the Entrepreneurship Scale and the Enterprise Innovation Performance Scale, as shown in Figure 9.

In Figure 9A, Q1–Q10 on the abscissa represent the 10 items of the entrepreneurship scale. In Figure 9B, Q1–Q6 on the abscissa represent the 6 items of the enterprise innovation performance scale. In Figure 9, the observed variable factor analysis results of innovative collective entrepreneurship are 0.596, 0.643, and 0.706, respectively. The observed variable factor analysis results of risk-taking entrepreneurship are 0.759, 0.637, and 0.652, respectively. The observed variable factor analysis results of Integrity entrepreneurship are 0.642, 0.589, and 0.730, respectively. The standardized parameter estimates of all measurement items are greater than 0.5, indicating that the Entrepreneurship Scale has good convergent validity.

### Correlation and Regression Analysis of Entrepreneurship and Technological Innovation Performance

**Figure 10:**
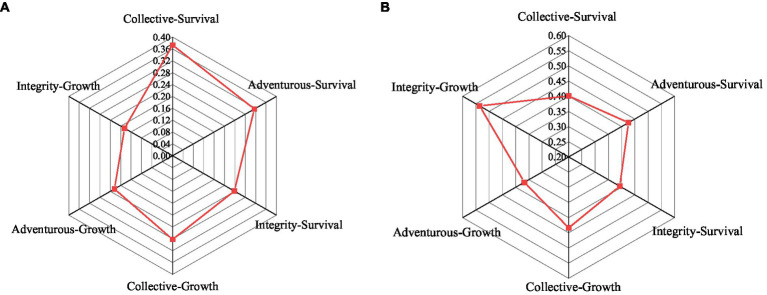
Analysis of entrepreneurship and technological innovation performance. **(A)** Correlation analysis of entrepreneurship and technological innovation performance and **(B)** Regression analysis of entrepreneurship and technological innovation performance.

Regression analysis and correlation analysis are carried out for each dimension of independent variable entrepreneurship and each dimension of dependent variable new venture performance, as shown in Figure 10.

In Figure 10A, the correlation coefficients between independent variables innovative spirit, adventurous spirit, integrity, and enterprise survival performance are 0.401, 0.426, and 0.393, respectively, which are positive correlations. The correlation coefficients between innovative spirit, adventurous spirit, integrity, and enterprise survival performance are 0.434, 0.367, and 0.536, respectively. There is a significant and positive correlation. In Figure 10B, after adding the independent variable entrepreneurship based on the regression of the control variable, the three dimensions of entrepreneurship, collective innovation, risk-taking, and integrity have a significant positive impact on the innovation survival performance of enterprises.

### Results Discussion

Questionnaires are used to collect relevant data information. The influence degree of each dimension of entrepreneurship on enterprise innovation performance is further studied. Firstly, the distribution of the selected companies in this survey is analyzed statistically. Among them, 31.3% of the enterprises are established for no more than 5 years, 24.7% are established between 5 and 10 years, 18% are established between 10 and 15 years, and 26% are established between 15 and 15 years. Small-scale enterprises accounted for 64.7%, and large enterprises (1,001 employees and above) accounted for 35.3%. Enterprises are mainly concentrated in private enterprises, accounting for 55%. Joint-stock listed companies account for 42.3%, and limited liability companies account for 36.3%. The sample distribution is relatively even.

Secondly, the designed scale is analyzed for reliability and validity. The results show that the scale has very good internal consistency and convergent validity. Finally, the correlation between entrepreneurship and technological innovation performance is analyzed by regression. The results show a significant positive correlation between innovative entrepreneurship, risk-taking entrepreneurship, aggressive entrepreneurship, and enterprise survival performance and growth performance. The three dimensions of entrepreneurship will all impact the entrepreneurial performance of enterprises, and the degree of impact is different. These works go further based on previous research and are more instructive for the development of enterprises.

## Conclusion

In the existing research, scholars usually regard each dimension of entrepreneurship to study its impact on the innovation performance of enterprises and have not conducted in-depth research on whether each dimension has an impact on the innovation performance of enterprises. This study innovatively explores the influence of various dimensions of entrepreneurial spirit on each dimension of corporate innovation performance. Firstly, concepts and measures of entrepreneurship and innovation performance are elaborated. Secondly, correlation hypotheses between variables are proposed. Finally, questionnaires are designed to test hypotheses. Finally, through statistical software to analyze the survey results, the conclusion is drawn: there is a positive correlation between innovative entrepreneurship, risk-taking entrepreneurship, Integrity entrepreneurship, and enterprise survival performance. There was also a significant positive correlation between these spirits and growth performance. Entrepreneurship and its three dimensions have a significant positive impact on entrepreneurial performance. The disadvantage is that due to limited energy. The questionnaires are mainly concentrated in the eastern coastal areas where the economy is relatively developed, and the market development is more mature. The quality of entrepreneurs of innovative enterprises in the central and western regions is not involved. These factors may also affect the final study results. Results of studies across regions are not analyzed comparatively. In the future, the institutional environment and corporate strategy of the enterprise will be further studied. The results of studies across regions will be further analyzed for comparative analysis. This study examines the logical relationship and impact mechanism between entrepreneurs’ entrepreneurship and corporate innovation performance, and it has a certain guiding role in China’s innovative enterprise management practices. Entrepreneurship is integrated into the corporate culture and daily management, which plays an important and positive role in the innovation capability and performance improvement of the company in the context of digital transformation.

## Data Availability Statement

The raw data supporting the conclusions of this article will be made available by the authors, without undue reservation.

## Ethics Statement

The studies involving human participants were reviewed and approved by Wuhan Polytechnic University Ethics Committee. The patients/participants provided their written informed consent to participate in this study. Written informed consent was obtained from the individual(s) for the publication of any potentially identifiable images or data included in this article.

## Author Contributions

All authors listed have made a substantial, direct, and intellectual contribution to the work, and approved it for publication.

## Conflict of Interest

The authors declare that the research was conducted in the absence of any commercial or financial relationships that could be construed as a potential conflict of interest.

## Publisher’s Note

All claims expressed in this article are solely those of the authors and do not necessarily represent those of their affiliated organizations, or those of the publisher, the editors and the reviewers. Any product that may be evaluated in this article, or claim that may be made by its manufacturer, is not guaranteed or endorsed by the publisher.
